# Decreased mebrofenin uptake in patients with non-colorectal liver tumors requiring liver volume augmentation—a single-center analysis

**DOI:** 10.1007/s00423-024-03280-5

**Published:** 2024-03-11

**Authors:** M. H. Fard-Aghaie, L. Stern, T. Ghadban, I. Apostolova, W. Lehnert, S. Klutmann, T. Hackert, J. R. Izbicki, J. Li, P. H. von Kroge, A. Heumann

**Affiliations:** 1 Department of General, Visceral and Thoracic Surgery, University Medical Center Hamburg-Eppendorf, Martinistr. 52, 20246 Hamburg, Germany; 2 Department of Diagnostic and Interventional Radiology and Nuclear Medicine, University Medical Center Hamburg-Eppendorf, Martinistr. 52, 20246 Hamburg, Germany

**Keywords:** PHLF, Liver augmentation, Hepatobiliary scintigraphy, Extended liver resection

## Abstract

**Background:**

Posthepatectomy liver failure (PHLF) remains a life-threatening complication after hepatectomy. To reduce PHLF, a preoperative assessment of liver function is indispensable. For this purpose, ^99m^Tc-mebrofenin hepatobiliary scintigraphy with SPECT (MSPECT) can be used. The aim of the current study was to evaluate the predictive value of MSPECT for PHLF in patients with non-colorectal liver tumors (NCRLT) compared to patients with colorectal liver metastasis (CRLM) undergoing extended liver resection.

**Methods:**

We included all patients undergoing extended liver resections via two-stage procedures between January 2019 and December 2021 at the University Medical Center Hamburg-Eppendorf, Germany. All patients received a preoperative MSPECT.

**Results:**

Twenty patients were included. In every fourth patient, PHLF was observed. Four patients had PHLF grade C. There were no differences between patients with CRLM and NCRLT regarding PHLF rate and future liver remnant (FLR) volume. Patients with CRLM had higher mebrofenin uptake in the FLR compared to those with NCRLT (2.49%/min/m^2^ vs. 1.51%/min/m^2^; *p* = 0.004).

**Conclusion:**

Mebrofenin uptake in patients with NCRLT was lower compared to those patients with CRLM. However, there was no difference in the PHLF rate and FLR volume. Cut-off values for the mebrofenin uptake might need adjustments for different surgical indications, surgical procedures, and underlying diseases.

## Background

Liver surgery is still a mainstay of therapy in primary and secondary liver tumors. Perioperative techniques lead to a significant rise in indications and improved safety in hepatobiliary (HPB) surgery [1]. However, one of the most concerning issues in HPB surgery remains posthepatectomy liver failure (PHLF) due to low future liver remnant (FLR). For extended resections, several techniques to augment liver volume have been described. Besides classical portal vein embolization (PVE) [2], associating liver partition and portal vein ligation for staged hepatectomy (ALPPS) has emerged as a potent tool for two-stage procedures [3]. Several adjustments of this technique have significantly decreased postoperative mortality [4].

However, liver volume as conventionally measured with computed tomography cannot distinguish between liver volume and liver function and may therefore underestimate volume/function mismatch [5]. This discrepancy can be exacerbated if there has been a previous liver injury (e.g., chemotherapy).

One tool to overcome this drawback and correctly assess liver function is the ^99m^technetium-mebrofenin hepatobiliary scintigraphy with SPECT (MSPECT) [6, 7]. MSPECT uses ^99m^Tc radio-labeled mebrofenin, an acid, to estimate regional and global liver function, as opposed to indocyanine green and LiMax Tests which cannot differentiate regional inequalities in hepatic function [8]. In 2010, a cut-off value of 2.69%/min/m^2^ in the FLR has been proposed for safe extended liver resections [6]. In 2020, Tomassini et al. were able to confirm this value in 98 patients treated by ALPPS [9].

Although some encouraging data has been presented in the literature, MSPECT and its implications on a heterogeneous cohort requiring extended resections with various liver volume augmentation techniques have not been published yet. In the current literature, ALPPS in patients with non-colorectal liver tumors (NCRLT) is associated with higher morbidity and mortality compared to colorectal liver metastasis (CRLM) [10]. Therefore, we aimed to elucidate the difference in liver function in patients with CRLM and NCRLT assessed by MSPECT undergoing extended liver resection.

## Material and methods

### Study design and study population

This single-center retrospective study included all patients that were treated at the University Medical Center Hamburg-Eppendorf between January 2019 and December 2021 and patients who met all the following criteria:

1. Age ≥ 18 years
2. Extended liver resections via two-stage procedures for CRLM or NCRLT
3. Preoperative estimation of the liver function by ^99m^Tc-mebrofenin hepatobiliary scintigraphy with SPECT as part of clinical routine

### Data collection and surgical procedure

Clinical and perioperative data regarding treatment and disease characterization were collected from the patient’s electronic medical records.

These included age-modified Charlson comorbidity score, administration of perioperative chemotherapy or monoclonal antibodies until 12 weeks prior to surgery, and presence of preoperative hyperbilirubinemia defined as a serum level > 2 mgl/dl. Additionally, we included the ALBI score to assess the preoperative liver function [11].

The quality of the liver parenchyma was categorized into fibrosis, steatosis, and normal histology. The specimens were retrieved in the step 2 operation.

Two senior surgeons (J.L. and A.H.) performed all operations. For resection, all patients required liver volume augmentation due to inadequate preoperative FLR. These included the ALPPS procedure (either classical, partial, or hybrid), portal vein embolization (PVE), hepatic vein ligation (HVL), a combination of HVL and PVE, and two-stage-hepatectomy supported by PVE. The type of liver resection was reported utilizing the Brisbane classification [12]. We investigated the interval between the first operation/intervention and the second step of the approach and the interval between the end of the chemotherapy (if applied) and MSPECT. For this purpose, we categorized patients with an interval “chemotherapy to MSPECT” > 8 weeks and < 8 weeks. Furthermore, the type of liver augmentation, the clearance of the FLR at the first step, vascular resections, and additional resections were noted.

Data collection was performed in accordance with local legal requirements (§ Hamburgisches Krankenhausgesetz). The study was approved by the Medical Ethical Committee approval number: PV3548, Hamburg, Germany. All procedures performed in this study involving human participants were in accordance with the ethical standards of the institutional and national research committee and with the 1964 Helsinki Declaration and its later amendments or comparable ethical standards.

### ^99m^Tc-mebrofenin hepatobiliary scintigraphy with SPECT

The technique of MSPECT-imaging as described by Rassam et al. [13] was used.

All MSPECTs were obtained after the first operation or intervention, respectively. The interval between the first operation/intervention and the date of the last MSPECT was taken into consideration (usually 14 days).

**Fig. 1:**
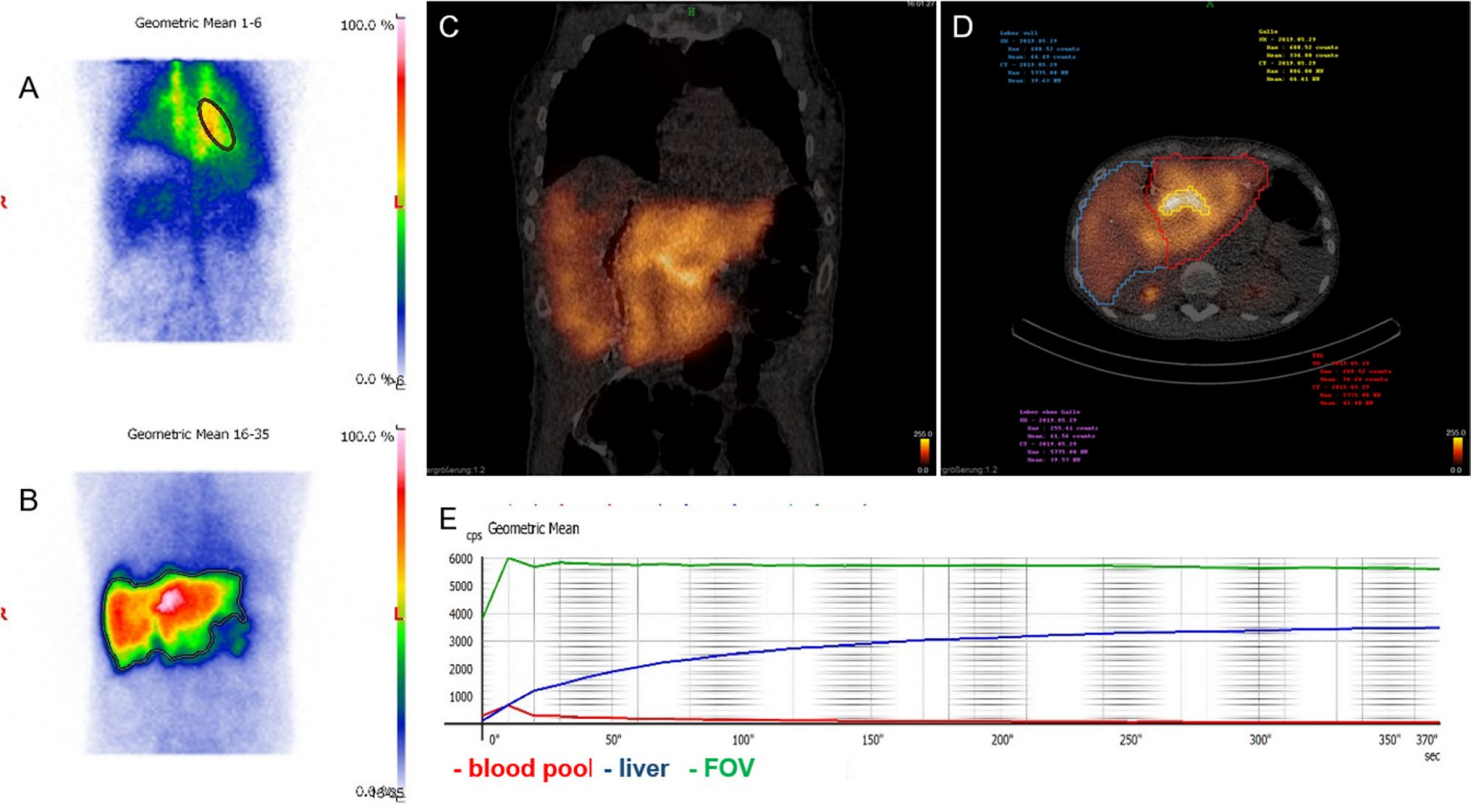
Clinical example of a patient (female, 56 years) with hepatic metastasis of colorectal cancer. **A** Geometric mean of the early frames of the first dynamic sequence depicting the ROI of the left ventricle. **B** Geometric mean image of the first dynamic sequence (150–350 s) with ROI of the total liver. **C** Coronal slice of the SPECT CT fusion image depicting the mebrofenin uptake in the liver after ALPPS-procedure. **D** Transversal slice of the fused SPECT and CT after segmentation of the total liver, FRL, and intrahepatic bile ducts. **E** Time-activity curves of the first dynamic scan for blood pool, liver, and whole field-of-view used for calculation of MUR

Mebrofenin SPECT was performed using a triple-head camera (Mediso AnyScan Trio) equipped with LEHRHS collimators. The planar dynamic acquisition from the anterior and posterior view was started immediately after an intravenous bolus injection of approximately 200 MBq ^99m^Tc-mebrofenin (ROTOP-EHIDA). A fast dynamic sequence was acquired with 38 frames of 10 s frame duration followed immediately by a SPECT with 60 frames of 8 s frame duration and additional low dose CT (100 kV, 30 mAs) for attenuation correction. The second dynamic sequence was started immediately after the SPECT/CT (20 frames of 60 s/frame). Image processing and quantification were performed as extensively described by Rassam et al. In short, a geometric mean dataset was generated using the dynamic sequences (Fig. 1A, B). Regions of interest (ROI) were defined in the first dynamic sequence for the generation of time-activity curves (TAC) of the liver, left ventricle and whole field of view (Fig. 1A, B). Using these TAC, the mebrofenin uptake rate (MUR) was calculated using the formula by Ekman et al. [14]. The calculated MUR in the percentage of the total administered Tc-99m-mebrofenin activity imbibed by the liver per minute was scaled by the body surface area. The functional distribution of the counts of the future liver remnant was determined in SPECT/CT after segmentation of the total liver and future liver remnant (InterView™ FUSION Processing Software, MEDISO) (Fig. 1C, D). For delineation of FLR, recent contrast-enhanced imaging together with the low dose CT was used to define anatomical landmarks (Fig. 1D). The activity within the intrahepatic bile ducts was replaced by average counts of the adjacent liver parenchyma. A ratio of the FLR counts and total liver counts as a percentage of total liver function was used to express the FLR function. Additionally, the biliary excretion rate of FLR in %/min was determined from the second dynamic scan using the described method [13].

We used the cut-off value of 2.69%/min/m^2^ as proposed by de Graaf et al. [6] to assess PHLF. Additionally, the average values for the mebrofenin uptake rate of the FLR were compared, and clinically relevant groups were stratified accordingly.

### Endpoints

The primary endpoint of this study was the incidence of PHLF. For the assessment, we used the classification by the International Study Group of Liver Surgery (ISGLS) [15]. As secondary endpoints, we assessed the morbidity rates, which were graded according to the Clavien-Dindo classification [16]. In addition, the overall mortality defined as death within 90 days after surgery was recorded.

### Statistical analysis

For data analysis, Statistical Package for Social Sciences statistical software (SPSS©) version 27.0 (IBM Corp. Released 2020. IBM SPSS Statistics for Macintosh, Armonk, NY: IBM Corp) was used.

Categorial variables are expressed as numbers (%). Continuous values are presented as median with range. The Kolmogorov-Smirnov-test was used to verify the normal distribution of the datasets. Correlation of categorial data was tested by Fisher’s exact test. Continuous data were tested with Student’s *t* test or Mann-Whitney *U* test as appropriate. We used the Kruskal-Wallis test to assess the significance of more than two categories. A two-sided *p*-value < 0.05 was considered statistically significant.

## Results

### Patient characteristics

Between January 2019 and December 2021, we identified 20 patients who met the aforementioned inclusion criteria. The median age of the cohort was 63.5 (40–82) with predominately male patients (65%). The median Charlson score was seven (3–10).

To further investigate differences or correlations, patients were stratified according to their diagnosis (CRLM vs. NCRLT). Twelve patients suffered from NCRLT. These included intrahepatic cholangiocarcinoma (1), perihilar cholangiocarcinoma (4), hepatocellular carcinoma (2), gallbladder carcinoma (1), and others (3). The median age was 67.5 (40–82) and the median Charlson score was 6 (3–10).

Eight patients underwent hepatectomy for CRLM of which all were treated with preoperative chemotherapy including seven patients receiving additional monoclonal antibody therapy (bevacizumab and panitumumab, respectively). In seven patients, preoperative hyperbilirubinemia was present. The median age was 58.5 (50–69) and the median Charlson score was 7 (7–9).

Table 1 depicts all demographic and perioperative data stratified according to the diagnosis. More patients in the CRLM group received preoperative chemotherapy and monoclonal antibody therapy (both *p* = < 0.0001). Age, gender, preoperative hyperbilirubinemia, and the Charlson score did not statistically differ between both groups. Extended resections were more evident in the NRCLT group (*p* = 0.019).

**Table 1:** Demographic and perioperative data stratified according to the diagnosis

	All (*n* = 20)	CRLM (*n* = 8)	NCRLT (*n* = 12)	*p*-value
Age, median (range)	63.5 (40–82)	58.5 (50–69)	67.5 (40–82)	0.175
Gender, male, *n* (%)	13 (65)	4 (50)	9 (75)	0.356
Preoperative chemotherapy*, *n* (%)	8 (60)	8 (100)	-	**< 0.001**
Preoperative antibody therapy°, *n* (%)	7 (35)	7 (87.5)	-	**< 0.001**
Preoperative hyperbilirubinemia, *n* (%)	7 (35)	1 (12.5)	6 (50)	0.158
Age-modified Charlson Comorbidity Index (range)	7 (3–10)	7 (7–9)	6 (3–10)	0.076

*NCRLT* non-colorectal liver tumors, *CRLM* colorectal liver metastasis

*Including FOLFOX, FOLFIRI, FOLFIRINOX, and FOLFOXIRI

°Including bevacizumab and panitumumab

The ALBI score was not different between both groups (*p* = 0.106). The histopathological examination regarding the quality of the liver parenchyma revealed fibrosis and steatosis in five patients, respectively. The mebrofenin uptake was not influenced by the quality of the liver parenchyma (*p* = 0.118).

### Patients’ outcome

Type of hypertrophy-induction, vascular resections, and other simultaneous resections were not different between groups as shown in Table 2.

**Table 2:** Operative data of patients stratified according to their diagnosis

	All (*n* = 20)	CRLM (*n* = 8)	NCRLT (*n* = 12)	*p*-value
Surgical procedure, *n* (%)				0.019*
Right hepatectomy Extended right hepatectomy Extended left hepatectomy Left lateral resection^++^ Others	6 (30) 11 (55) 1 (5) 1 (5) 1 (5)	4 (50) 2 (25) - 1 (12.5) 1 (12.5)	2 (16.7) 9 (75) 1 (8.3) - -	
Type of hypertrophy induction, *n* (%)				0.158
ALPPS Other°	13 (65) 7 (35)	7 (87.5) 1 (12.5)	6 (50) 6 (50)	
Vascular resection, *n* (%)				0.550
Yes No	5 (25) 15 (75)	- 8 (100)	5 (41.7) 7 (58.3	
Other simultaneous resections, *n* (%)				1.000
Yes No	5 (25) 15 (75)	2 (25) 6 (75)	3 (25) 9 (75)	
FLR after augmentation, ml, median (range)	741.5 (380–1390)	727.0 (514–1130)	775.0 (380–1390)	0.395
FLR after augmentation, %, median (range)	0.50 (0.25–0.75)	0.53 (0.31–0.74)	0.48 (0.25–0.75)	0.371
Clearance of FLR, *n* (%)				**0.028**
Yes No	11 (55) 9 (45)	7 (87.5) 1 (12.5)	4 (33.3) 8 (66.7)	
Mebrofenin uptake, %/min/m^2^, median (range)	1.90 (0.73–3.66)	2.49 (1.60–3.66)	1.51 (0.73–2.64)	**0.004**
Morbidity according to Clavien-Dindo, *n* (%)				1.000
< 3A > 3A	8 (40) 12 (60)	3 (37.5) 5 (62.5)	5 (41.7) 7 (58.3)	
PHLF according to ISGLS, *n* (%)				0.603^+^
None A B C	15 (75) 1 (5) - 4 (20)	7 (87.5) - - 1 (12.5)	8 (66.7) 1 (8.3) - 3 (25)	
Mortality, *n* (%)	3 (15)	1 (12.5)	2 (16.7)	1.000

*ALPPS* associating liver partition and portal vein ligation for staged hepatectomy, *FLR* future liver remnant, *ISGLS* International Study Group of Liver Surgery, *PHLF* posthepatectomy liver failure

*Extended resections and non-extended resections were pooled

°Including portal vein embolization and liver vein ligation

^+^All patients with PHLF pooled and tested against all patients without PHLF

^++^Prior right hepatectomy performed in this patient

Overall mortality and morbidity rates (above Clavien-Dindo 3A) amounted to 15% and 60% in all patients. The cause of mortality of all three patient was PHLF.

In 25% of all patients, PHLF was observed. Four patients had PHLF grade C. None of the postoperative outcomes differed significantly between the CRLM- and NCRLT-group (*p* = 1.000, *p* = 1.000, and *p* = 0.603) (Table 2).

Next, PHLF, morbidity (above Clavien-Dindo 3A) and mortality were each correlated to the administration of preoperative chemotherapy and antibodies, clearance of the FLR, vascular resections, other simultaneous resections, and presence of preoperative hyperbilirubinemia. A significant correlation was only observed between PHLF and preoperative hyperbilirubinemia (*p* = 0.031). A longer interval between the first operation/intervention and sequential two-stage resection did not translate into fewer rates of PHLF (*p* = 0.161).

### Liver function assessed by mebrofenin uptake in patients with NCRLT and CRLM

**Fig. 2:**
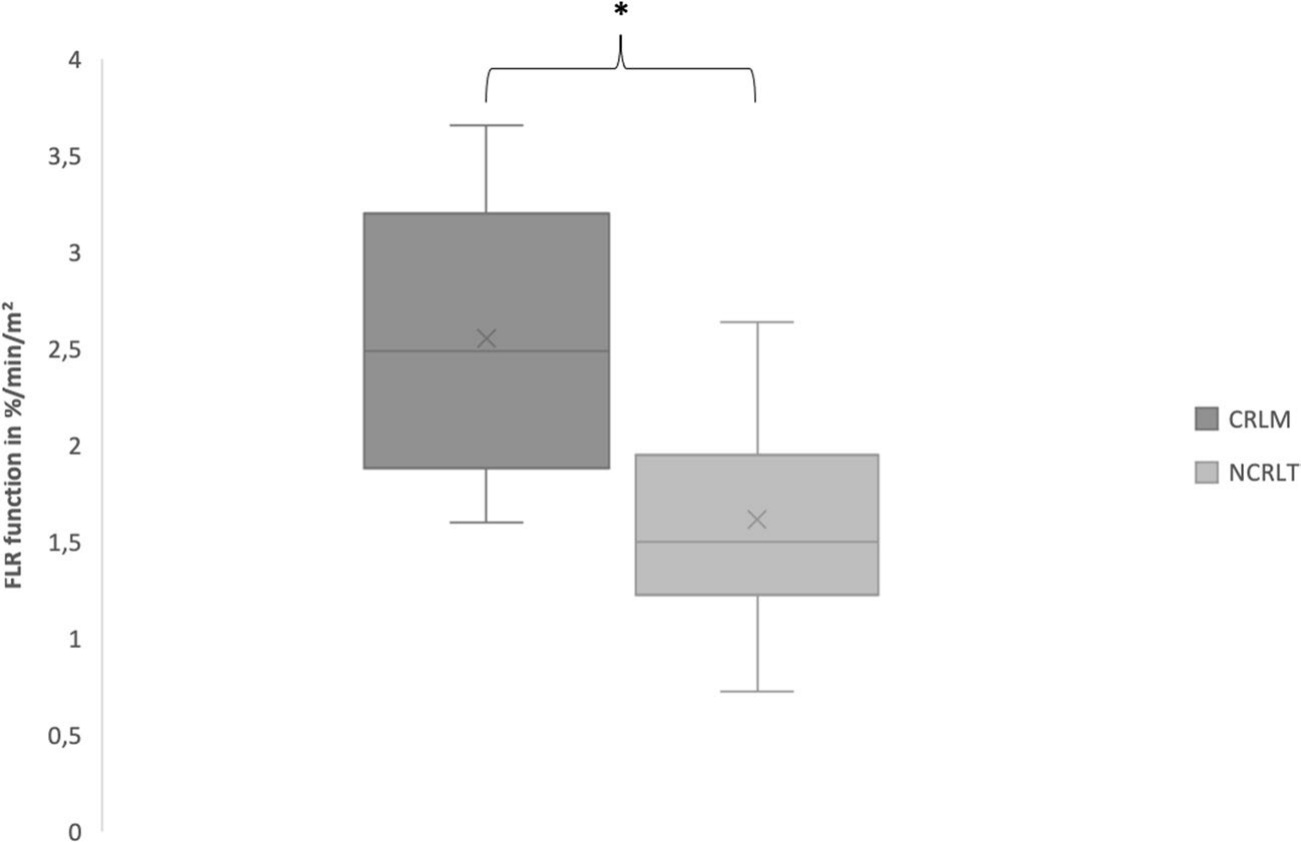
The function of the future liver remnant (FLR) assessed by ^99m^Tc-mebrofenin hepatobiliary scintigraphy with SPECT of both groups after augmentation is shown in a box plot. The difference in patients with colorectal liver metastasis (CRLM) and patients with non-colorectal liver tumors (NCRPT) reaches significance (* = *p* < 0.005) while FLR showed no statistically significance between both groups (*p* > 0.05)

Patients with CRLM had significantly higher mebrofenin uptake in the FLR compared to those suffering from NCRLT (2.49%/min/m^2^ vs. 1.51%/min/m^2^; *p* = 0.004) (Fig 2). More patients in the CRLM cohort reached the cut-off value of 2.69%/min/m^2^ (4 out of 8). None of the patients in the NCRLT group reached this value. The difference was statistically significant (*p* = 0.014).

In patients with CRLM, an interval of more or less than 8 weeks between chemotherapy and MSPECT did not show any statistical significance (*p* = 0.556).

A reached cut-off value of 2.69%/min/m^2^ did not translate into less PHLF statistically (*p* = 1.00). Furthermore, neither mortality nor morbidity (above Clavien-Dindo 3A) was associated with the cut-off value of 2.69% (*p* = 0.509 and *p* = 1.000).

There was no statistical difference in uptake between male and female patients (1.82%/min/m^2^ vs. 2.31%/min/m^2^, *p* = 0.176). Additionally, no difference between deceased and alive patients regarding the uptake was found (2.00%/min/m^2^ vs. 1.99%/min/m^2^; *p* = 0.984).

## Discussion

In this current study, mebrofenin uptake in the FLR was significantly lower in the NCLRT cohort than in the CRLM group while FLR showed no significant differences between both groups. More patients in the CRLM group reached the reported cut-off value of 2.69%/min/m^2^. However, decreased uptake did not translate into statistically significant mortality, morbidity, or PHLF rates between both groups. This outcome must be interpreted with caution as in 2020 the international multicenter study by Tomassini et al. [9] was able to show that in 98 patients, a cut-off of < 2.7%/min/ m^2^ was associated with a higher risk of PHLF after ALPPS step 1.

Although PHLF rates did not differ statistically, more grade B and C PHLFs were evident in the NCLRT group. This can be explained by the more extensive resections performed in the NCLRT cohort (*p* = 0.019). Additionally, more vascular resections were performed in this group, although the difference did not reach statistical significance. A longer time interval between hypertrophy induction and hepatectomy was not associated with lower PHLF. This is in line with the results of Ribero et al. [17] demonstrating the major hepatic hypertrophy in the first 21 days after induction and subsequent plateau phase. Moreover, more patients in the CRLM group received chemotherapy with antibodies prior to surgery, and the FLR was more subjected to clearance in this group without increasing morbidity or mortality. The only patient suffering from severe PHLF with subsequent demise in the CRLM group was preoperatively treated by a stent placement due to obstructive hyperbilirubinemia. This correlates with the elaborated association between preoperative hyperbilirubinemia and higher rates of PHLF in this current study.

Although hyperbilirubinemia was more present in the NCLRT group, no statistical difference was found compared to the CRLM group (CRLM: *n* = 1 vs. NCLRT: *n* = 6, *p* = 0.158). Simultaneously, more patients in the NCLRT group suffered from PHLF, also without reaching a statistically significant difference between groups.

Bilirubin and mebrofenin are known for their competitive interaction [8], and therefore, MSPECT might underestimate liver function [18]. In our study, uptake was higher in patients without preoperative hyperbilirubinemia, although no significant data was obtained (2.18%/min/m^2^ vs. 1.63%/min/m^2^; *p* = 0.127).

Olthof and colleagues [18] were able to calculate a cut-off of 8.5%, which resulted in a negative predictive value of 94% for PHLF in patients with perihilar cholangiocarcinoma. This is in accordance with the current study that hyperbilirubinemia has detrimental effects on the liver consecutively leading to PHLF. The effect of impaired liver regeneration in obstructive jaundice is well known in the literature [19].

In contrast to Olthof et al., our patients’ collective already received hypertrophy induction using different techniques. Nonetheless, the suggested cut-off values have not been reached in all cases. As the resection of biliary tumors remains the only curative therapeutic modality, the resection was carried out anyway. Regarding the NCLRT cohort, all patients with PHLF grade B/C were treated with either ALPPS (1×) or sequential HVL and PVE (2×) due to an intrahepatic cholangiocarcinoma, perihilar cholangiocarcinoma, and Gallbladder carcinoma. In the CRLM group, one patient treated with Hybrid-ALPPS with preoperative obstructive jaundice died due to severe PHLF, although the cut-off value of 2.7%/min/m^2^ was reached.

Increased rates of PHLF in ALPPS for indications other than CRLM have been reported [20]. Additionally, the influence of HVL before PVE for liver volume augmentation on PHLF remains unclear. The combination of NCRLT, ALPPS, and HVL/PVE and preoperative hyperbilirubinemia might be responsible for the fatal outcome in these patients.

There are several limitations in this study that need to be considered. The results in this study are constrained by its retrospective design leading to a heterogenous population with variable therapies and indications. Furthermore, as described above, the small sample size might be responsible for insufficient statistical power to reproduce previously published data.

Mebrofenin uptake in patients with NCRLT was statistically lower compared to those patients with CRLM but did not correlate with higher or lower rates of PHLF and FLR volume. Therefore, cut-off values for the uptake in the FLR might need adjustments for different surgical indications, surgical procedures, and underlying diseases.

## References

[1] Dokmak S, Fteriche FS, Borscheid R, Cauchy F, Farges O, Belghiti J (2013). 2012 Liver resections in the 21st century: we are far from zero mortality. HPB (Oxford).

[2] Narula N, Aloia TA (2017). Portal vein embolization in extended liver resection. Langenbeck's Arch Surg.

[3] Schnitzbauer AA, Lang SA, Goessmann H, Nadalin S, Baumgart J, Farkas SA (2012). Right portal vein ligation combined with in situ splitting induces rapid left lateral liver lobe hypertrophy enabling 2-staged extended right hepatic resection in small-for-size settings. Ann Surg.

[4] Raptis DA, Linecker M, Kambakamba P, Tschuor C, Muller PC, Hadjittofi C (2019). Defining benchmark outcomes for ALPPS. Ann Surg.

[5] Truant S, Baillet C, Deshorgue AC, El Amrani M, Huglo D, Pruvot FR (2017). Contribution of hepatobiliary scintigraphy in assessing ALPPS most suited timing. Updat Surg.

[6] de Graaf W, van Lienden KP, van Gulik TM, Bennink RJ (2010). (99m)Tc-mebrofenin hepatobiliary scintigraphy with SPECT for the assessment of hepatic function and liver functional volume before partial hepatectomy. J Nucl Med.

[7] Rassam F, Olthof PB, van Lienden KP, Bennink RJ, Erdmann JI, Swijnenburg RJ (2020). Comparison of functional and volumetric increase of the future remnant liver and postoperative outcomes after portal vein embolization and complete or partial associating liver partition and portal vein ligation for staged hepatectomy (ALPPS). Ann Transl Med.

[8] Gupta M, Choudhury PS, Singh S, Hazarika D (2018). Liver functional volumetry by Tc-99m mebrofenin hepatobiliary scintigraphy before major liver resection: a game changer. Indian J Nucl Med.

[9] Tomassini F, D'Asseler Y, Linecker M, Giglio MC, Castro-Benitez C, Truant S (2020). Hepatobiliary scintigraphy and kinetic growth rate predict liver failure after ALPPS: a multi-institutional study. HPB (Oxford).

[10] Linecker M, Bjornsson B, Stavrou GA, Oldhafer KJ, Lurje G, Neumann U (2017). Risk adjustment in ALPPS is associated with a dramatic decrease in early mortality and morbidity. Ann Surg.

[11] Johnson PJ, Berhane S, Kagebayashi C, Satomura S, Teng M, Reeves HL (2015). Assessment of liver function in patients with hepatocellular carcinoma: a new evidence-based approach-the ALBI grade. J Clin Oncol.

[12] Strasberg SM, Belghiti J, Clavien PA, Gadzijev E, Garden JO, Lau WY (2000). The Brisbane 2000 terminology of liver anatomy and resections. HPB..

[13] Rassam F, Olthof PB, Richardson H, van Gulik TM, Bennink RJ (2019). Practical guidelines for the use of technetium-99m mebrofenin hepatobiliary scintigraphy in the quantitative assessment of liver function. Nucl Med Commun.

[14] Ekman M, Fjalling M, Holmberg S, Person H (1992). IODIDA clearance rate: a method for measuring hepatocyte uptake function. Transplant Proc.

[15] Rahbari NN, Garden OJ, Padbury R, Brooke-Smith M, Crawford M, Adam R (2011). Posthepatectomy liver failure: a definition and grading by the International Study Group of Liver Surgery (ISGLS). Surgery..

[16] Dindo D, Demartines N, Clavien PA (2004). Classification of surgical complications: a new proposal with evaluation in a cohort of 6336 patients and results of a survey. Ann Surg.

[17] Ribero D, Abdalla EK, Madoff DC, Donadon M, Loyer EM, Vauthey JN (2007). Portal vein embolization before major hepatectomy and its effects on regeneration, resectability and outcome. Br J Surg.

[18] Olthof PB, Coelen RJS, Bennink RJ, Heger M, Lam MF, Besselink MG (2017). (99m)Tc-mebrofenin hepatobiliary scintigraphy predicts liver failure following major liver resection for perihilar cholangiocarcinoma. HPB (Oxford).

[19] Yokoyama Y, Nagino M, Nimura Y (2007). Mechanism of impaired hepatic regeneration in cholestatic liver. J Hepato-Biliary-Pancreat Surg.

[20] Balci D, Sakamoto Y, Li J, Di Benedetto F, Kirimker EO, Petrowsky H (2020). Associating liver partition and portal vein ligation for staged hepatectomy (ALPPS) procedure for cholangiocarcinoma. Int J Surg.

